# Verschwörungsdenken, Wahn und Virtualität

**DOI:** 10.1007/s11757-022-00717-9

**Published:** 2022-05-18

**Authors:** Thomas Fuchs

**Affiliations:** Karl-Jaspers-Professur für Philosophie und Psychiatrie, Psychiatrische Universitätsklinik, Ruprecht-Karls-Universität Heidelberg, Voßstr. 4, 69115 Heidelberg, Deutschland

**Keywords:** Verschwörungstheorien, Reduktion von Komplexität, Zufall, Subjektzentrierung, Virtualisierung, Conspiracy theories, Reduction of complexity, Contingency, Subject centrism, Virtualization

## Abstract

„Nichts ist, wie es scheint“, „nichts geschieht zufällig“, und „alles ist miteinander verbunden“ – diese drei Grundannahmen charakterisieren Verschwörungstheorien unterschiedlicher Provenienz. Damit zeigen sie eine deutliche Parallelität zur Struktur von paranoidem Bedeutungserleben und Wahn. Dem stehen jedoch wichtige Unterschiede gegenüber, etwa die Bildung ausgedehnter Gruppen von Verschwörungsgläubigen, denen keine paranoiden „Wahngemeinschaften“ entsprechen. Der Aufsatz untersucht zunächst beide Phänomene vergleichend und wendet sich dann der Frage zu, wie die Virtualisierung der Kommunikation in den sozialen Medien die Bildung von verschwörungstheoretischen Gemeinschaften begünstigt.

## Abstract (en)

“Nothing is as it seems”, “nothing happens by chance”, and “everything is connected”—These three basic assumptions characterize conspiracy theories of different provenance. Thus, they show a clear parallelism to the structure of paranoid and delusional experience. However, this is contrasted with important differences, such as the formation of extended groups of conspiracy believers, to which no paranoid “delusional communities” correspond. The paper first examines both phenomena comparatively and then turns to the question of how the virtualization of communication in social media fosters the formation of conspiracy theory communities.

Bill Gates hat das Coronavirus entwickeln lassen, um die Weltbevölkerung zu reduzieren und an Impfstoffen Geld zu verdienen. In seinem Auftrag implantiert die Bundesregierung mit der Impfung den Menschen Mikrochips, um ihre Gedanken zu steuern (Lamberty und Nocun 2021). Eine internationale pädophile Elite verschleppt Kinder, um aus ihrem Blut eine Verjüngungsdroge zu gewinnen. Die Wahl des US-Präsidenten wurde von Agenten des Deep State gefälscht und damit Trump gestohlen, um dessen heroischen Kampf gegen die Kinderschänder zu sabotieren; und so weiter.

Solche und andere Verschwörungstheorien, aber auch weniger systematisierte „Fake News“ und „alternative Fakten“ haben in den vergangenen beiden Pandemiejahren eine besondere Bedeutung und Beachtung erlangt. Sie sind freilich kein neues Phänomen: Schon nach einer Untersuchung der Friedrich-Ebert-Stiftung aus dem Jahr 2017/2018 glaubten 45,7 % der Befragten, dass geheime Organisationen wahrscheinlich großen Einfluss auf politische Entscheidungen haben (Zick et al. 2019); 32,7 % waren der Meinung, dass Politiker und andere Führungspersönlichkeiten „nur Marionetten dahinterstehender Mächte“ seien. In einer Studie der Konrad-Adenauer-Stiftung waren schon vor der Coronakrise 11 % der Befragten *sicher* von einer Weltverschwörung überzeugt („Es gibt geheime Mächte, die die Welt steuern“); der Anteil hat seither nicht zugenommen (Roose 2020).

Unabhängig von ihrer Verbreitung stellt sich aus psychiatrischer Sicht die Frage, ob solchen Weltsichten ein paranoider Denkstil oder – im Falle bizarrer Überzeugungen wie der von blutdürstigen Kinderschändern oder Reptilienmenschen – gar ein krankhafter Erklärungswahn zugrunde liegen könnte. Träfe das zu, müsste man den Anhängern von Verschwörungstheorien wohl eher eine psychiatrische Behandlung anbieten als eine argumentative Auseinandersetzung. Die oft angenommene Analogie von Verschwörungstheorien und Wahn erscheint zwar schon angesichts der vergleichsweise geringen Prävalenz von schizophrenen Erkrankungen (ca. 1 % der Bevölkerung) ganz unangemessen. Dennoch ist die Abgrenzung zwischen beiden Phänomenen aufgrund vielfältiger Ähnlichkeiten und Überlagerungen nicht selbstverständlich.

Ich werde im folgenden skizzenhaften Überblick zunächst einige Gemeinsamkeiten und Unterschiede zwischen Verschwörungsideen und paranoidem Wahn darstellen und mich dann der Frage zuwenden, welche Strukturen des Internets und der virtuellen Medien die Verbreitung von Verschwörungsdenken begünstigen. Auch diese Strukturen haben nämlich, wie sich zeigen wird, gewisse Analogien mit Formen des paranoiden Erlebens.

## Verschwörung und Wahn – Ähnlichkeiten und Unterschiede

„Nichts ist, wie es scheint“, „nichts geschieht durch Zufall“, und „alles ist hängt mit allem zusammen“: Durch diese drei Grundannahmen werden „conspiracy theories“ beispielsweise in der gegenwärtigen amerikanischen Politologie charakterisiert (Barkun 2013; Butter 2018). Schon eine schlichte Geste wie die berühmte „Raute“ von Angela Merkel erscheint dann nicht mehr als einfache Handbewegung, sondern nimmt Zeichencharakter an, für etwas Hintergründiges, vielleicht die Zugehörigkeit zu einem Geheimbund oder Ähnliches. In der Konsequenz kann es dann nur noch um das Ziel gehen, dieses „Dahinter“, die eigentlichen Bedeutungen und Agenten zu entschlüsseln. Die Drahtzieher werden häufig durch die Denkfigur des „cui bono?“ ausgemacht: Wem nützt es, wer hat einen Vorteil davon?

Nun finden sich diese Merkmale in analoger Form auch im paranoiden Wahnerleben; Klosterkötter (2020) weist auf die „weitreichende Übereinstimmung in der Erlebnisstruktur, im Wahrheitsanspruch und in der mutmaßlichen Entlastungsfunktion“ bei beiden Phänomenen hin. So sind der Ausschluss des Zufalls und der Zeichencharakter der wahrgenommenen Umwelt bekannte Charakteristika des Wahns (Berner 2013). Auch die unerschütterliche Gewissheit und Unkorrigierbarkeit vieler Verschwörungsgläubiger unterscheidet sie häufig nicht von Wahnkranken. Gleiches gilt für die Bizarrheit des Inhalts, etwa wenn Personen wie Benedikt XVI., Queen Elisabeth, Angela Merkel oder Hillary Clinton in Wahrheit Reptilienwesen sein sollen, die die Menschheit versklaven wollen.

Vergleichbar ist auch die *Funktion* der beiden Phänomene, nämlich die Herstellung von Orientierung, Sinn und Kohärenz in einer unüberschaubar und unkontrollierbar gewordenen Welt. Verschwörungsideen blühen immer dann auf, wenn Unsicherheit, Ängste und Zweifel an etablierten Systemen wachsen und traditionelle Deutungsmuster nicht mehr greifen (Butter 2018). Sie entspringen dem Bedürfnis, eine undurchschaubare, als bedrohlich empfundene Krisenlage erklärbar zu machen, ihre Komplexität zu reduzieren und ihre schwer erträgliche Zufälligkeit zu bewältigen, indem sie als intentional verursacht gedeutet wird. Ähnlich erfüllt der Wahn die Funktion, der rätselhaften, unheimlichen und bedrohlichen Wahrnehmungswelt der vorausgehenden Wahnstimmung einen neuen, kohärenten (Wahn‑)Sinn zu verleihen.

Was beispielsweise in coronabezogenen Verschwörungstheorien verleugnet wird, ist offensichtlich die Kontingenz, also die Zufälligkeit und Unvorhersehbarkeit, mit der ein unsichtbares Virus plötzlich zu einer weltweiten tödlichen Gefahr geworden ist. Die resultierende Verunsicherung und Desorientierung führen zu dem Versuch, ein kohärentes Weltbild wiederherzustellen, nämlich durch eine umfassende Umdeutung der Realität: Die anfängliche Erfahrung von Kontingenz und Ohnmacht kehrt sich um in die Gewissheit, die geheimen Machenschaften der Mächtigen durchschaut zu haben. Es ist eben nicht zufällig, was da in unserem Land geschieht, sondern wohlgeplant, hinterhältig organisiert und heimtückisch ausgeführt. So können im Geheimen wirkende Personen oder Gruppen als Verantwortliche eines Geschehens angesehen werden, das sonst kontingent und undurchschaubar bliebe. Diese vermeintliche Einsicht bietet nicht zuletzt Möglichkeiten zur Gegenwehr, und damit stellt sie die eigene Handlungsfähigkeit und Kontrolle zumindest bis zu einem gewissen Grad wieder her.^1^

Die Funktion der neuen Realitätsdeutung ist also sowohl im Verschwörungsglauben als auch im Wahn die Reduktion einer bedrohlichen Hyperkomplexität. Um diese Entlastungswirkung nicht zu gefährden, immunisieren sich die Betroffenen im weiteren Verlauf systematisch gegen widersprechende Evidenzen. Wahnkranke schotten sich gegen alle Zweifelseinwände ab, indem sie die Perspektivenübernahme blockieren oder ihre Erfahrungen als schlichtweg einzigartig betrachten. Verschwörungsgläubige ihrerseits „entlarven“ anderslautende Informationen als gezielte Täuschungen der Eliten oder als „Fake News“.

Damit sind einige übereinstimmende Strukturen von Verschwörungsideen und Wahnerleben benannt; der paranoide Denkstil ist ihnen beiden gemeinsam. Auf der anderen Seite bestehen jedoch auch deutliche *Unterschiede. *Sieht man einmal davon ab, dass der Wahn ohnehin im Rahmen einer psychischen Krankheit mit meist vielfältigen anderen, typischen Symptomen auftritt, so unterscheidet er sich von Verschwörungsideen vor allem durch einen Verlust von Intersubjektivität und eine kommunikativ geschlossene Struktur (Fuchs 2020a):

- *Isolierende Wirkung des Wahns*. Wahn bedeutet grundsätzlich einen Verlust der Fähigkeit zum Perspektivenabgleich, der die Kommunikation mit anderen über das Wahnthema weitgehend verunmöglicht und – sieht man von der sehr seltenen Folie-à-deux ab – niemals zu einer „Wahngemeinschaft“ führt. Dies steht in deutlichem Gegensatz zu Verschwörungstheorien, die durchaus mit anderen geteilt werden, ja ansteckend wirken können. In der Regel kommt es zu intensiver Kommunikation mit Gleichgesinnten, zur Ausbildung einer Gruppenidentität, etwa im Sinne eines gemeinsamen Opferstatus und zu einer hohen Gruppenkohäsion besonders in den Echokammern der sozialen Medien. Da diese zugleich hohe Prominenz versprechen, kann es gerade bei bekannten Verschwörungstheoretikern auch um die Befriedigung besonders ausgeprägter Geltungsbedürfnisse gehen. Narzisstische Befriedigung verleiht auch das Bewusstsein, zu den besonderen Individuen zu gehören, die im Gegensatz zu den „Schafen“ der Mehrheitsgesellschaft die verborgenen Machenschaften der Eliten durchschaut haben: „Wir sind die Wissenden.“
- *Subjektzentrierung des Wahns*. Ganz anders verhält es sich in paranoiden Psychosen, wo sich eine ausgesprochene Subjektzentrierung des Erlebens erkennen lässt, sodass nur die *eigene Person* als Ziel der Handlungen oder Manipulationen anderer betrachtet wird. Es ist gerade der Subjektzentrismus, die häufig so genannte *Truman-Show*-Symptomatik (Madeira et al. 2016), die den hochgradig angsterfüllten Charakter des paranoid-psychotischen Erlebens bedingt. Im Wahn bezieht sich die Bedrohung oder Verfolgung immer nur auf das betroffene Individuum, nicht auf gesellschaftliche Ereignisse. Anders bei Verschwörungstheorien, die in der Regel eine Wir-Gruppe oder sogar eine ganze Gesellschaft als Opfer verborgener Machenschaften betrachten. Nocun und Lamberty beschreiben den Unterschied mit der eingängigen Formel: „Während paranoide Menschen glauben, dass praktisch jeder hinter ihnen her ist, denken Verschwörungsideologen, dass ein paar mächtige Menschen hinter fast jedem her sind“ (Nocun und Lamberty 2020, S. 34).

## Virtualisierung und Verschwörung

Bei einem phänomenologischen Vergleich zwischen Wahnerleben und Verschwörungsideen lassen sich also strukturelle Übereinstimmungen, aber auch deutliche Unterschiede bestimmen. Untersuchen wir nun, wie die Struktur der Virtualität in den digitalen Medien die Entstehung und Verbreitung von Verschwörungsideen begünstigt.

Zunächst erzeugen digitale Medien grundsätzlich ambivalente, also halb-reale, halb-virtuelle Erfahrungsräume, in denen sich die Unterschiede zwischen Sein und Schein, Original und Simulation, realer und virtueller Präsenz immer mehr auflösen. Die fortschreitende Entkörperung der Wahrnehmung und das Auftreten von „Phantomen“ (Anders 1956) oder „Simulakren“ (Baudrillard 1982), also Zwittergebilden zwischen Realität und Fiktion, führen dazu, dass der Realitätsbezug in technologisch vermittelten Umwelten grundsätzlich labiler wird (Fuchs 2020b). Man denke nur an die zahlreichen Zuschauer, die bei der Geburt eines Babys in der TV-Dauerserie *Lindenstraße* tatsächlich Babykleider und Spielzeug an die Fernsehsender schicken.

Zugleich werden wir gegenwärtig zunehmend Zeugen einer gezielten Herstellung verzerrter oder fingierter Realitäten im Interesse politischer Gruppen und Machthaber, die sich dazu der digitalen Medien bedienen. Oft weiß man nicht mehr, ob sich Ereignisse tatsächlich so abgespielt haben, wie Bilder und Berichte suggerieren, und der „Faktencheck“ ist Ausdruck des ständigen Kampfes um Realitätsdeutungen. Trotz aller Bemühungen um Wahrheit weist die Ausbreitung von Parallelwelten in den sozialen Netzwerken darauf hin, dass die fundamentale Unterscheidung von Faktum und Fiktum zunehmend infrage gestellt ist. Die Wirklichkeit gilt oft selbst nur noch als ein Cluster von Daten oder Informationen, das willkürlich zusammengestellt oder konstruiert werden kann. Nicht umsonst stammt ja auch der Begriff der „alternativen Fakten“ von der ersten Pressesprecherin Donald Trumps.

Die entstehenden Parallelwelten haben inzwischen bildkräftige Namen erhalten, nämlich „Echokammern“ und „Filterblasen“. Betrachten wir kurz die beiden Phänomene:

- *Echokammern* sind homogene Netzwerke von Nutzern, in denen Meinungen durch stetige Wiederholung innerhalb eines geschlossenen Systems bestätigt und verstärkt werden. Informationen, die den eigenen Erwartungen entsprechen, werden also selektiert, widersprechende Informationen ausgeblendet.
- *Filterblasen* entstehen durch automatisierte, algorithmisierte Angleichung von Webseiten und Informationen an die Erwartungen und Gewohnheiten der Nutzer, etwa in sozialen Netzwerken (Pariser 2011). Natürlich können auch politische Interessengruppen technische Möglichkeiten wie Bots und Algorithmen gezielt zur Beeinflussung nutzen. So entsteht ein spezifisch auf die Rezipienten zugeschnittener Informationskosmos.

Beide Phänomene treten häufig parallel auf, da Filterblasen Echokammereffekte noch verstärken können. Insgesamt führen sie zum gleichen Resultat, nämlich zu einer *Fragmentierung der klassischen Öffentlichkeit*, in der man sich prinzipiell noch über eine gemeinsame Realität verständigen konnte. Aufgrund der zunehmenden Virtualisierung ohne die Möglichkeit der Überprüfung und ohne konkrete, verkörperte Interaktion mit anderen gehen also die geteilten lebensweltlichen Grundlagen für Wahrheitsansprüche verloren. Damit entstehen Überzeugungsgemeinschaften für ganz heterogene Interpretationen der Wirklichkeit.

Hinzu kommt, dass das Internet heute „Cyberkaskaden“ von Informationen erzeugt, die sich extrem rasch entwickeln und daher nur schwer zu kontrollieren sind. Dafür wird zunehmend die Metapher der *Viralität* verwendet, also einer Art „Ansteckung“, wobei zu beachten ist, dass die Beteiligten die Online-Inhalte häufig nicht einfach nur weiterleiten, sondern auch verändern und umdeuten. Die viralen Informationen können sich so nach dem Muster der „stillen Post“ inhaltlich weiterentwickeln und neue Varianten generieren – in Analogie zur aktuellen Coronapandemie.

Als letzter Aspekt sei schließlich die *Hyperkomplexität des Virtuellen* genannt. Das Internet ermöglicht eine universelle Vernetzung bei gleichzeitiger Undurchschaubarkeit seiner Strukturen. Alles erscheint mit allem verbunden, ohne dass sich diese Verbindungen nachvollziehen lassen; anonyme Agenten und Unternehmen steuern den Informationsfluss. Dies findet eine Entsprechung in der Wahnstimmung in beginnenden Psychosen, wo alles Unauffällige im Hintergrund für die Betroffenen salient und zugleich verdächtig wird, während umgekehrt alles Vordergründige sich in bloßen Anschein oder Täuschung verwandelt. So entsteht schließlich der überwältigende Eindruck, die Realität sei nur eine von anonymen Mächten inszenierte Vorstellung (Fuchs 2005). Auch wenn wir gesehen haben, dass Verschwörungstheorien keine Wahnphänomene darstellen, erkennen wir hier doch noch einmal die Gemeinsamkeit, nämlich die *Reduktion von Komplexität*, die der Wahn ebenso wie die Verschwörungstheorien leisten, jeweils auf ihre unterschiedliche Weise.

## Resümee

Verschwörungsideen weisen, wie wir sahen, eine Reihe von Gemeinsamkeiten mit paranoiden Wahnideen auf. Dazu gehören (a) die Überzeugung von einer verborgenen Verbindung vordergründig eher unauffälliger Ereignisse; (b) der Ausschluss des Zufallsprinzips, das sich aufdrängende Zusammenhänge neutralisieren könnte, und (c) die Annahme einer geheimen Intentionalität von „Drahtziehern“ oder mächtigen Gruppen, die die Geschehnisse steuern. In einer Ausgangssituation, die durch massive Verunsicherung und Desorientierung gekennzeichnet ist, führt das vermeintliche Durchschauen der verborgenen Zusammenhänge zu einer signifikanten psychischen Entlastung und Restabilisierung. Die Funktion der neuen Realitätsdeutung ist also in beiden Fällen die Reduktion einer bedrohlichen Hyperkomplexität. Um diese Funktion nicht zu gefährden, immunisieren sich die Betroffenen systematisch gegen korrigierende Evidenzen, die entweder konsequent ausgeblendet oder als „Fake News“ diskreditiert werden.

Auf der anderen Seite bestehen die wesentlichen Unterschiede zwischen Wahn und Verschwörungsideen (a) in der isolierenden Subjektzentrierung, die die Welt des Wahnkranken kennzeichnet, und (b) in seinem Unvermögen, über seine paranoiden Überzeugungen in Kommunikation und Austausch mit Gleichgesinnten zu treten. Die Unfähigkeit zur Perspektivenübernahme, die den Wahn charakterisiert (Fuchs 2020a), lässt eine „Wahngemeinschaft“ nicht zu, während Verschwörungsgläubige sich in virtuellen „communities“ oder realen Begegnungen wechselseitig bestätigen und aus der Beachtung durch andere sogar hohen narzisstischen Gewinn ziehen können.

Die Virtualisierung der Kommunikation und Information im digitalen Zeitalter begünstigt solche Überzeugungsgemeinschaften, etwa in Form von „Echokammern“ und „Filterblasen“. Generell hat der Rückgang von realen Interaktionen und verkörperter Realitätserfahrung eine zunehmende Nivellierung der Unterscheidung von Realität und Virtualität zur Folge, die in verschiedenen Bereichen der Gesellschaft erkennbar wird. Verschwörungstheorien stellen eine bedeutsame Erscheinungsform dieser Derealisierung dar und gehen mit der Gefahr einer Fragmentierung der demokratischen Öffentlichkeit einher. In ihnen manifestiert sich nicht zuletzt die Überforderung der Individuen durch Hyperkomplexität der medial vermittelten Welten, der sie durch einen Rückzug in Echokammern von Gleichgesinnten begegnen, um so ihre infrage gestellte Identität zu stabilisieren. Die Psychopathologie vermag gewisse strukturelle Gemeinsamkeiten, aber auch bedeutsame Unterschiede von Verschwörungsglauben und paranoiden Wahnideen feststellen und so dazu beitragen, diese für unsere Gesellschaft bedeutsamen Phänomene besser zu verstehen.

## Footnotes

1 Dass sich diese Handlungsbereitschaft nicht auf verbale Proteste beschränken muss, ist inzwischen deutlich geworden. Die bereits erwähnte Studie der Friedrich Ebert-Studie wies einen signifikanten Zusammenhang zwischen Verschwörungsneigungen und Gewaltbereitschaft nach (Zick et al. 2019).
